# Pyocyanin-Enhanced Neutrophil Extracellular Trap Formation Requires the NADPH Oxidase

**DOI:** 10.1371/journal.pone.0054205

**Published:** 2013-01-14

**Authors:** Balázs Rada, Meghan A. Jendrysik, Lan Pang, Craig P. Hayes, Dae-goon Yoo, Jonathan J. Park, Samuel M. Moskowitz, Harry L. Malech, Thomas L. Leto

**Affiliations:** 1 Laboratory of Host Defenses, National Institute of Allergy and Infectious Diseases, National Institutes of Health, Rockville, Maryland, United States of America; 2 Department of Infectious Diseases, College of Veterinary Medicine, University of Georgia, Athens, Georgia, United States of America; 3 Department of Pediatrics, Massachusetts General Hospital, Boston, Massachusetts, United States of America; 4 Department of Pediatrics, Harvard Medical School, Boston, Massachusetts, United States of America; University of Alabama-Birmingham, United States of America

## Abstract

Beyond intracellular killing, a novel neutrophil-based antimicrobial mechanism has been recently discovered: entrapment and killing by neutrophil extracellular traps (NETs). NETs consist of extruded nuclear DNA webs decorated with granule proteins. Although NET formation is an important innate immune mechanism, uncontrolled NET release damages host tissues and has been linked to several diseases including cystic fibrosis (CF). The major CF airway pathogen *Pseudomonas aeruginosa* establishes chronic infection. Pseudomonas imbedded within biofilms is protected against the immune system, but maintains chronic inflammation that worsens disease symptoms. Aberrant NET release from recruited neutrophils was found in CF, but the underlying mechanisms remain unclear. One of the most important Pseudomonas virulence factors is pyocyanin, a redox-active pigment that has been associated with diminished lung function in CF. Here we show that pyocyanin promotes NET formation in a time- and dose-dependent manner. Most CF Pseudomonas clinical isolates tested produce pyocyanin *in vitro*. Pyocyanin-derived reactive oxygen species are required for its NET release. Inhibitor experiments demonstrated involvement of Jun N-terminal Kinase (JNK) and phosphatidylinositol 3-Kinase (PI3K) in pyocyanin-induced NET formation. Pyocyanin-induced NETs also require the NADPH oxidase because NET release in chronic granulomatous disease neutrophils was greatly reduced. Comparison of neutrophils from gp91phox- and p47phox-deficient patients revealed that pyocyanin-triggered NET formation is proportional to their residual superoxide production. Our studies identify pyocyanin as the first secreted bacterial toxin that enhances NET formation. The involvement of NADPH oxidase in pyocyanin-induced NET formation represents a novel mechanism of pyocyanin toxicity.

## Introduction

Polymorphonuclear neutrophil granulocytes (PMN) provide the first line of defense against bacteria. Neutrophils migrate to the site of infection, engulf and kill the invaders by exposing them to a variety of antimicrobial peptides, proteins and reactive oxygen species. Neutrophils also combat pathogens by a recently described novel mechanism, formation of neutrophil extracellular traps (NETs). NETs are composed of a DNA backbone decorated with histones and several antimicrobial neutrophil granule components: myeloperoxidase (MPO), lactoferrin, elastase and bactericidal/permeability-increasing protein. [1] NETs kill bacteria *in vitro* by ensnaring the microbes within high local concentrations of the neutrophils’ weaponry. Recent live imaging of *in vivo* NET formation in an acute bacterial skin infection model provided further evidence for the importance of NETs in immunity. [2] NET formation (NETosis) is a novel form of neutrophil cell death different from apoptosis or necrosis. [3], [4] The mechanisms triggering NETs are poorly understood and are under investigation. Bacteria (whole cells, LPS, pilus) or inflammatory mediators (IL-8, IFN I+II, C5a) have been reported to induce NETs. [5] Reactive oxygen species (ROS) produced by the phagocytic NADPH oxidase are essential for NET formation, since neutrophils of chronic granulomatous disease (CGD) patients are unable to release NETs in response to a variety of stimuli. [4] CGD neutrophils produce very little or no superoxide due to genetic deficiencies in any one of several components of the superoxide-producing NADPH oxidase enzyme complex [6].

NETs are a crucial part of antimicrobial innate immunity; however, accumulating evidence suggests that uncontrolled NET release can also correlate with disease severity. [7] Aberrant NET formation has already been implicated in a variety of diseases including systemic lupus erythematosus, autoimmune small-vessel vasculitis and cystic fibrosis (CF). [8]–[10] Cystic fibrosis is a common inherited life shortening disease among Caucasians. [11] The primary cause of the disease is a genetic defect in the cystic fibrosis transmembrane conductance regulator (CFTR) protein, a cAMP-regulated anion channel expressed in several organs including the airways, pancreas and sweat glands. [12] The absence of normal CFTR in airways leads to altered ion transport across epithelial cells, thicker mucus and hindered microbial mucociliary clearance giving rise to chronic bacterial infections. [13] The most common Gram-negative pathogen infecting the airways of CF patients is *Pseudomonas aeruginosa*. [11] The bacterium colonizes CF airways early in life and establishes chronic infection, which is the major cause of death in CF. [14] In healthy airways *Pseudomonas aeruginosa* hardly ever causes problems and neutrophils play an important role in clearance of Pseudomonas. [15] In CF airways, however, persistent Pseudomonas infections are characterized by biofilm growth, which protects bacteria from both opsonization and access of neutrophils to phagocytose and kill them. The established presence of bacteria maintains chronic inflammation resulting in mucus hypersecretion and robust neutrophil infiltration through production of virulence factors such as pyocyanin [13].

The exotoxin pyocyanin is an important virulence factor of *Pseudomonas aeruginosa;* its induction through quorum signaling correlates with the biofilm growth stage of the bacterium accounting for the high concentrations found in CF patients’ airways. [16]–[19] Pseudomonas virulence is seriously diminished in the absence of pyocyanin production in mouse models. [20] Pyocyanin concentrations in CF sputum samples correlate with decreased lung function and its rate of decline. [19] Although pyocyanin has a wide range of toxic effects, the proposed basis for its toxicity is production of superoxide anions and downstream ROS inside of cells by oxidizing NAD(P)H. [21]–[23] This depletion of intracellular NADPH reserves supporting intracellular oxidant production imposes oxidative stress on host cells. In neutrophils, pyocyanin has been shown to lower NADPH levels and inhibit killing of *Staphylococcus aureus,* but its effect on NET formation has never been studied [24], [25].

Pseudomonas embedded within biofilms in CF airways is well-protected against the attack of recruited neutrophils, which can release their antimicrobial load into the airway lumen and contribute to tissue damage. [26] Pulmonary function of CF patients (FEV_1_) has been negatively correlated with sputum concentrations of DNA, myeloperoxidase (MPO) and autoantibodies against bactericidal-permeability increasing protein, all of which are neutrophil components that could be derived from NETs. [27], [28] Human recombinant DNAse treatment has been shown to improve mucociliary clearance within CF patients’ airways. [29] Pyocyanin has been shown to promote DNA release from *Pseudomonas aeruginosa*. [30] NETs were detected in CF patients’ sputa. [10] However, induction of NETs by *Pseudomonas aeruginosa* has not been characterized to date and nothing is known about signaling mechanisms or virulence factors participating in Pseudomonas-stimulated NET formation.

Here, we aimed at characterizing pyocyanin-elicited NET formation in adherent neutrophils. We investigated the effects of pyocyanin at concentrations reported in CF airways [18], [19]. Our data identify pyocyanin as a novel NET inducer that requires the NADPH oxidase for its action. Our findings suggest that NET induction by pyocyanin contributes to the inflammatory conditions found in CF airways.

## Results

### Pyocyanin Induces NET Formation

**Figure 1:**
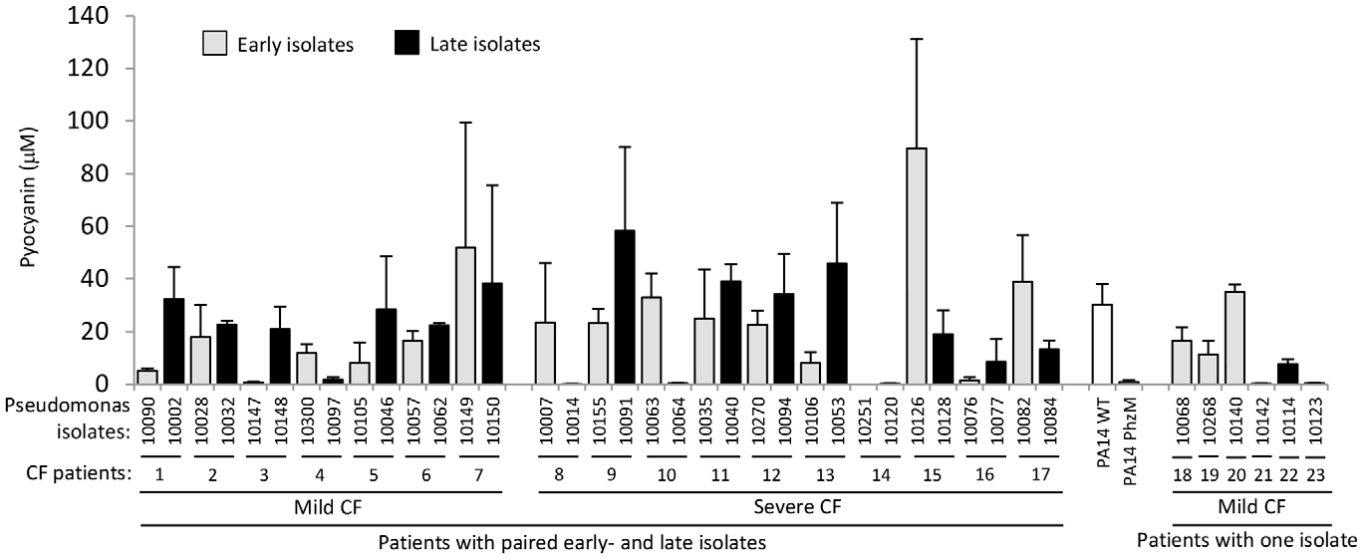
Majority of CF clinical isolates of *Pseudomonas aeruginosa* produce pyocyanin *in vitro*. Cystic fibrosis clinical isolates of *Pseudomonas aeruginosa* were grown in LB medium for 48 hrs and pyocyanin concentrations in the culture supernatants were determined. Data are organized according to disease severity of CF patients (mild/severe) or early/late phase origin of the isolates (for details see methods). Data show mean +/− S.E.M. of three independent experiments.

NET formation induced by bacteria requires ROS but it is unknown which microbial factor(s) mediate this process. [3] Since ROS originating from NADPH oxidase-independent sources (bolus H_2_O_2_, glucose oxidase (GO)) are capable of inducing NETs, we tested whether pyocyanin, a redox-active exotoxin of Pseudomonas, could affect NET formation. [4] Pyocyanin is essential for full virulence in a variety of Pseudomonas infection models. [15] One study found that sputa of three out of 4 CF patients contained pyocyanin at levels equal to or higher than those used in our study. [15] Another recent study reported high pyocyanin levels in CF patients’ airways which negatively correlated with lung function, clearly indicating that pyocyanin is an important contributor to CF airway pathology. [19] To assess the importance of pyocyanin production in Pseudomonas airway infection in CF we examined *in vitro* pyocyanin production by 40 CF clinical isolates of *Pseudomonas aeruginosa*. Pyocyanin concentrations were measured in the culture supernatants of stationary phase cultures and compared to the laboratory control strain PA14. (Fig. 1). Most of the clinical isolates produced pyocyanin, only six of the forty isolates did not produce the toxin (Fig. 1). Clinical isolates obtained from CF patients with mild or severe disease and obtained from early or late phases of the disease (from the same 17 patients) were compared. A wide range of production capabilities was detected from these isolates and no trends in pyocyanin production were observed. Average pyocyanin concentrations were (mean +/− SD, µM): mild CF (17.5+/−27.26), severe CF (24.2+/−31.01), early isolates (22.1+/−31.52), late isolates (19.7+/−26.57).

**Figure 2:**
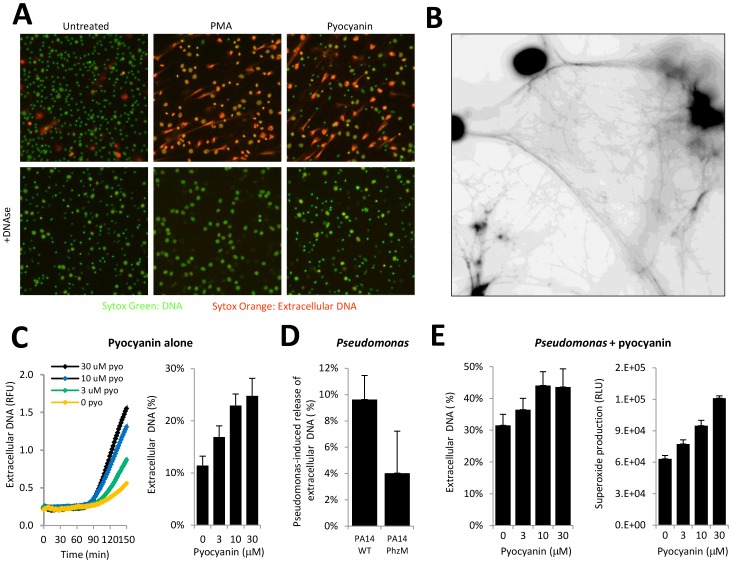
Pyocyanin induces extracellular DNA release in human neutrophils. A) Detection of NET formation in human neutrophils stimulated by pyocyanin. Adherent neutrophils were exposed to 20 µM pyocyanin or 100 nM PMA for 3 hours, stained with 2.5 µM Sytox Orange and 5 µM Sytox Green, and NETs were visualized by fluorescence microscopy. Samples in the lower panels were treated with 1 U/mL DNase1 (20 min 37C). Two other experiments resulted in similar data. B) Negative black and white image of pyocyanin-stimulated neutrophils reveals fine structural details of pyocyanin-triggered NETs. Adherent neutrophils were stimulated with 20 µM pyocyanin *in vitro* for 3 hrs, stained with DAPI and details of the NET structures were visualized by fluorescence microscopy. Results were converted to negative black and white images to achieve better contrast between DNA network and the background. This picture is representative of data obtained on 4 different donors. C) Quantification of NETs induced by pyocyanin. Neutrophils were incubated for 2.5 hrs with different concentrations of pyocyanin (0–30 µM) in the presence of Sytox Orange (2.5 µM). Release of extracellular DNA (increase in fluorescence) was followed in time with the fluorescence microplate assay (one representative experiment of six) (left panel). NET release was (increase in fluorescence in 2.5 hours) quantitated as percentage of maximal. Data represent mean +/−S.E.M. (n = 6) (right panel). D) Human neutrophils were exposed to 2×10^6^ wild-type (PA14 WT, pyocyanin-producing) and PhzM-deficient (PA14 PhzM, pyocyanin-deficient) *Pseudomonas aeruginosa* PA14 for 2.5 hours in the presence of 2.5 µM Sytox Orange. Increase in fluorescence induced by bacteria over baseline was calculated and expressed as increases in extracellular DNA (% of maximal) (mean+/−S.E.M., n = 4). E) Extracellular DNA release was initiated in adherent neutrophils by co-addition of wild-type *Pseudomonas aeruginosa* PA14 (2×10^6^/well) and increasing concentrations of purified pyocyanin (0,3,10,30 µM). Sytox Orange fluorescence (extracellular DNA release, 2.5 µM) and Diogenes chemiluminescence (superoxide production, integrated RLU/60 min) were increased in a dose-dependent manner by pyocyanin (mean+/−S.E.M., n = 4). PMA, phorbol 12-myristate-13-acetate.

**Figure 3:**
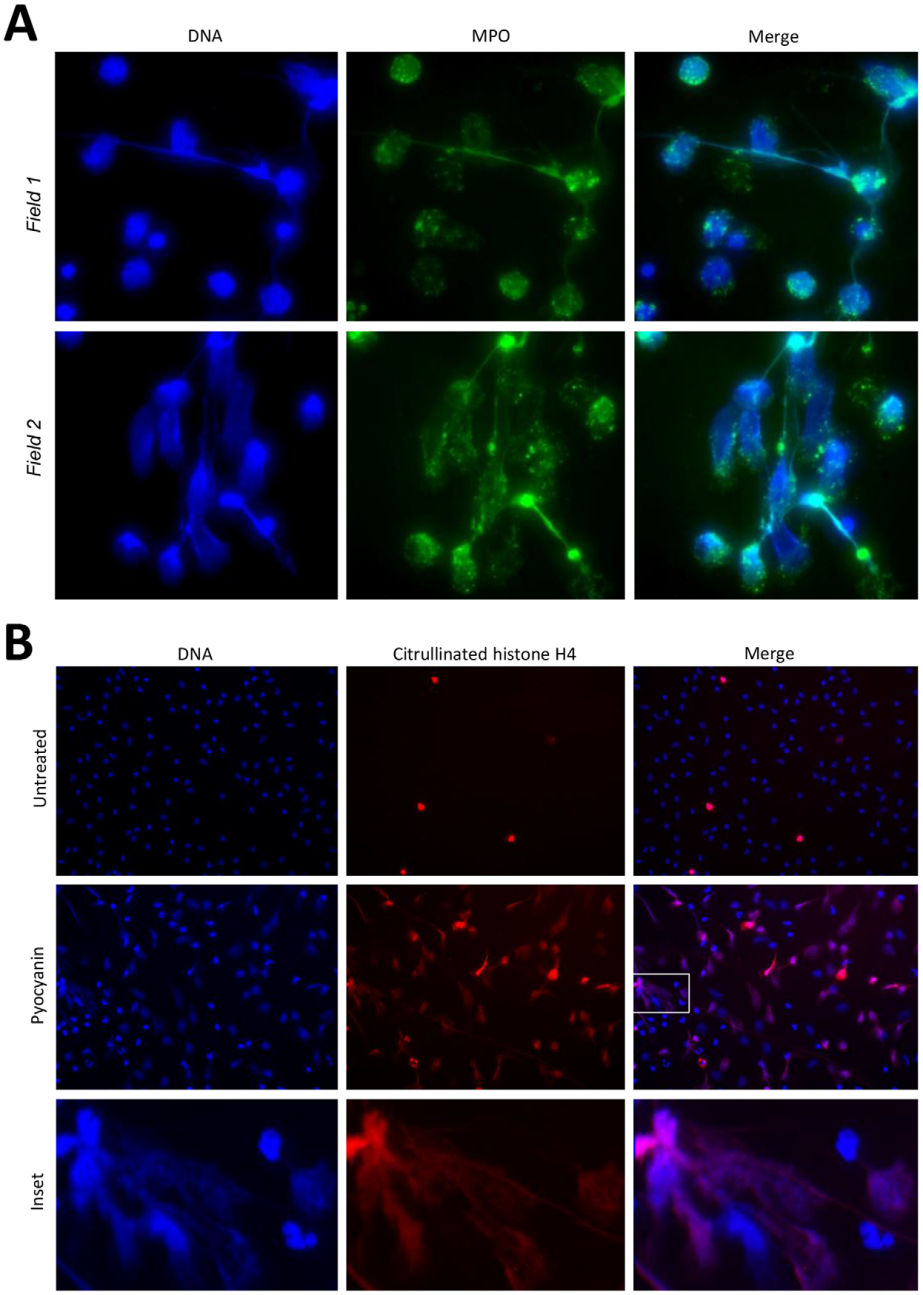
Myeloperoxidase and citrullinated histone H4 co-localize with extracellular DNA in pyocyanin-stimulated NETs. A) Human neutrophils were seeded on glass coverslips and incubated in the presence of 20 µM pyocyanin for 3 hours. Cells were fixed and stained for MPO (FITC-labeled anti-MPO Ab) and DNA (DAPI). Two independent fields show co-localization of MPO and DNA (one representative experiment, n = 3). B) Citrullinated histone H4 co-localizes with NET DNA structures in pyocyanin-stimulated neutrophils. Adherent neutrophils were treated with 20 µM pyocyanin for 3 hours, then fixed, washed and subjected to anti-citH4 immunostaining and DAPI staining. Two other experiments gave similar data.

Next, we examined whether the purified toxin itself is capable of inducing NETs. Adherent neutrophils were exposed to 20 µM pyocyanin for 3 hrs and cells were stained simultaneously with the membrane impermeable DNA dye Sytox Orange or the membrane-permeable DNA stain Sytox Green. We found that PMNs released NETs in response to pyocyanin (Fig.2A). Extracellular DNAse treatment degraded pyocyanin-induced NETs (Fig. 2A). A negative black and white image of DAPI-stained NETs in pyocyanin-treated PMNs reveals fine, detailed DNA structure (Fig. 2B). In a concentration range characteristic for CF patients’ airways (0–30 µM), pyocyanin induces NETs in a time- and dose-dependent manner (Fig. 2C). [18], [19] To appreciate the contribution of pyocyanin to NET formation induced by *Pseudomonas aeruginosa*, we compared NET release by the pyocyanin-secreting PA14 wild type strain and its pyocyanin (PhzM)-deficient mutant. Pyocyanin deficiency results in a 58.6+/−34.3% reduction in NET release induced by *Pseudomonas aeruginosa* (Fig. 2D). Furthermore, addition of the purified toxin to wild-type *Pseudomonas* increases NET release and superoxide production in a dose-dependent manner (Fig. 2E). In NETs granule components are attached to DNA fibers. [1] Deimination of certain arginine residues into citrullin in histones by peptidylarginine deiminase 4 (PAD4) has been shown to be required for NET formation. [31]–[33] To show co-localization of granule components and histones with DNA, we co-stained pyocyanin-treated neutrophils for myeloperoxidase (MPO) or citrullinated histone H4 (citH4) and DNA (DAPI). Both, MPO and citH4 staining co-localized with DNA staining in pyocyanin-treated PMNs (Fig. 3A–B).

### ROS are Required for Pyocyanin-triggered NETs

**Figure 4:**
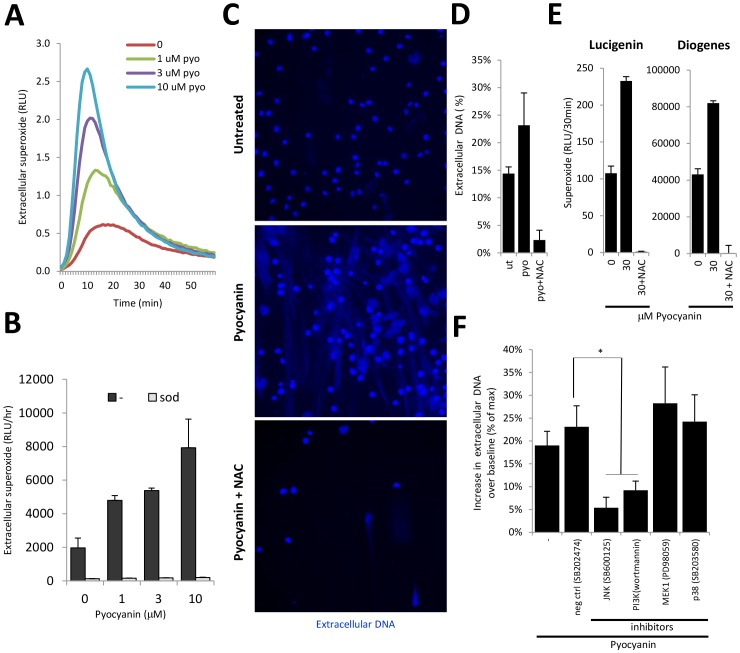
Pyocyanin-triggered NET formation involves reactive oxygen species, Jun N-terminal Kinase and Phosphatidylinositol 3-kinase. A) Representative kinetics of superoxide production in adherent human neutrophils exposed to 1, 3, 10 µM pyocyanin (Diogenes, 60 min, another donor gave similar data). B) Extracellular superoxide production stimulated by pyocyanin is dose-dependent and abolished by 12.5 µg/ml superoxide dismutase (sod) (mean+/−S.D. of two different donors, “–“ = untreated). C) NAC blocks pyocyanin-induced NET formation. Human neutrophils were pretreated with 10 mM NAC for 10 min followed by exposure to 20 µM pyocyanin for 3 hrs. Images of DAPI-stained NETs were taken with fluorescence microscopy. Similar results were obtained with another donor. D) Quantification of the inhibitory effect of NAC (10 mM) on pyocyanin-stimulated NET formation by the fluorescence (Sytox Orange) microplate assay. Data are mean+/− S.E.M. (n = 3). E) NAC (10 mM) blocks both basal and pyocyanin-elicited superoxide production in neutrophils (Lucigenin, Diogenes, mean+/−S.E.M., n = 3) F) Inhibitors of JNK (SB600125, 10 µM, p = 0.0124)) and PI3K (wortmannin, 100 nM, p = 0.0374) suppress pyocyanin-stimulated NET formation whereas p38 and MEK1 pathway inhibitors were without any effect (10µM PD980589, 10 µM SB203580). Data show mean+/− S.E.M. (n = 4). NAC, N-acetyl-cysteine; RLU, relative luminescence unit; JNK, Jun N-terminal Kinase; PI3K, Phosphatidylinositol 3-kinase; sod, superoxide dismutase; * marks significant changes were p<0.05.

Although pyocyanin has been shown to cause a broad range of toxic effects in different host cells, its diverse toxicity originates from production of ROS. [23] Pyocyanin lowers intracellular NADPH levels in neutrophils as it generates intracellular ROS. [24] We therefore asked if NETs induced by pyocyanin are mediated by ROS. Using the Diogenes-based superoxide detection method we found that pyocyanin enhanced superoxide release by adherent neutrophils in a dose-dependent manner (Fig. 4A, B). We next found that pretreatment of neutrophils with the ROS scavengers N-acetyl-cysteine (NAC, 10 mM in HBSS) blocked both spontaneous and toxin-elicited NET release (Fig. 4C, D). Both basal and pyocyanin-elicited superoxide productions were abolished by NAC pre-treatment (Fig. 4E). We conclude that ROS are required for pyocyanin to induce NETs.

### Jun N-terminal Kinase (JNK) and Phosphatidylinositol 3-kinase (PI3K) Mediate NET Release Induced by Pyocyanin

The signaling mechanisms behind NETosis are still largely unexplored although the Raf-MEK-ERK signaling pathway has already been implicated. [34] To gain insight into the signaling steps of pyocyanin-elicited NET formation, we surveyed different pathway inhibitors. We found that the JNK inhibitor (SB600125) and the PI3K inhibitor (wortmannin) exhibited strong inhibition (SB600125: 71.7%, wortmannin: 51.5%; mean, n = 4) on pyocyanin-stimulated NET release whereas inhibition of MEK1 and p38 had no significant effects (Fig. 4F).

### Pyocyanin Enhances Superoxide Production in Neutrophils by Activating the NADPH Oxidase

**Figure 5:**
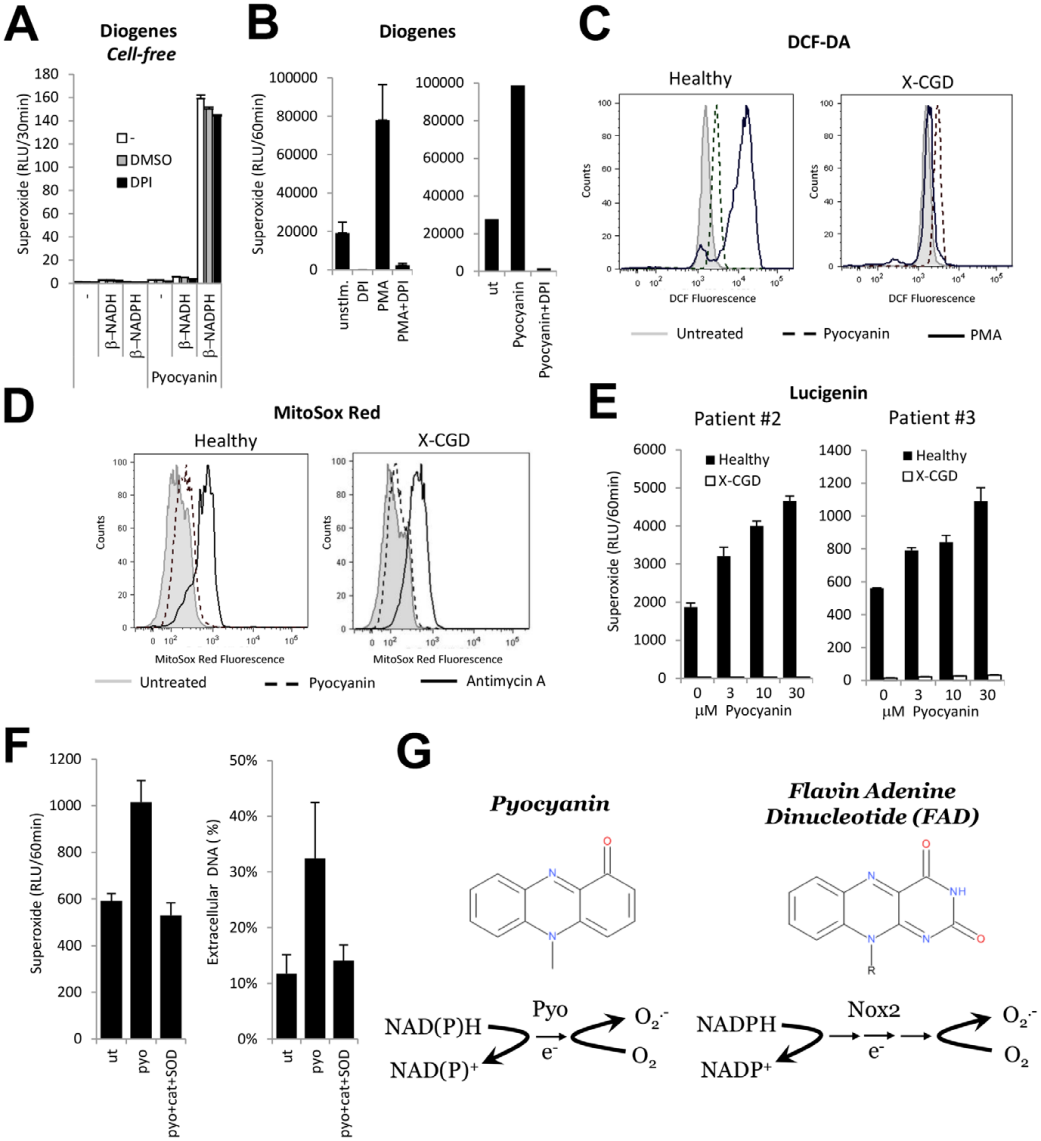
The NADPH oxidase is the source of extracellular superoxide mediating NET release in pyocyanin-stimulated human neutrophils. A) Pyocyanin (50 µM) oxidizes β-NADH (20 µM) and β-NADPH (20 µM) in a cell-free system and produces superoxide anions as detected by the Diogenes assay. DPI (10 µM) and DMSO (solvent of DPI) have no effect (data are mean+/− S.E.M., n = 3). B) DPI inhibits basal, PMA- and pyocyanin-stimulated superoxide production in human neutrophils. PMNs were pretreated with 10 µM DPI, stimulated by 20 µM pyocyanin and superoxide production was measured by the Diogenes assay for 60 min. Concentrations of PMNs were: 10^6^/mL for PMA stimulation and 5×10^6^/mL for pyocyanin. C) Pyocyanin produces low-level intracellular ROS in the absence of the NADPH oxidase. DCFDA-loaded healthy and X-CGD neutrophils (patient 1) were exposed to 20 µM pyocyanin or 100 nM PMA and intracellular production of reactive oxygen species was measured by flow cytometry. Similar results were obtained with patient 2′s cells. D) Low mitochondrial superoxide levels are detected in both, healthy and X-CGD neutrophils stimulated by pyocyanin. Healthy or CGD neutrophils (patient #2) loaded with MitoSox Red were exposed to 30 µM pyocyanin for 30 min and mitochondrial superoxide production was measured by flow cytometry. Data were only obtained from one CGD patient. Antimycin A was used as positive control. E) Dose-dependence of pyocyanin-stimulated superoxide production in normal and X-CGD neutrophils (Lucigenin) (patient 2 and 3). F) Scavenging extracellular ROS (catalase and SOD) eliminates pyocyanin-induced ROS production and NET formation in human neutrophils. Human neutrophils were exposed to 30 µM pyocyanin in the presence or absence of 1500 U/mL catalase and 12.5 µg/mL SOD. Superoxide production was followed with Lucigenin for 60 min; NET formation was followed for 4 hrs. Data represent mean+/− S.E.M. of two independent experiments. G) Similarity of chemical structures of pyocyanin and flavin adenine dinucleotide (FAD) and their redox reactions involved in superoxide generation. DMSO, dimethyl sulfoxide; DPI, diphenylene iodonium; RLU, relative luminescence unit; DCF-DA, 2′-7′-Dichlorodihydrofluorescein diacetate; CGD, chronic granulomatous disease; PMA, phorbol 12-myristate-13-acetate; PMN, polymorphonuclear neutrophil; FAD, flavin adenine dinucleotide; cat, catalase; SOD, superoxide dismutase.

Pyocyanin oxidizes reduced NADH or NADPH and produces superoxide under aerobic conditions (Fig. 5A). NAD(P)H oxidation by pyocyanin is insensitive to the NADPH oxidase inhibitor DPI in a cell-free *in vitro* system (Fig. 5A). In host cells, pyocyanin crosses the plasma membrane, oxidizes intracellular NAD(P)H pools and produces ROS. We therefore hypothesized that pyocyanin produces superoxide in PMNs in an NADPH oxidase-independent manner and that DPI would have no effect. The PMA-stimulated superoxide response was blocked by DPI as expected, but to our surprise DPI also entirely inhibited the pyocyanin-elicited response (Fig. 5B). This indicates that pyocyanin is acting through a flavoenzyme in intact PMNs, most probably the NADPH oxidase. To test if pyocyanin-enhanced superoxide production is NADPH oxidase-dependent, we compared normal and X-CGD neutrophils by using three different indicators. DCF-DA oxidation measures low amounts of intracellular ROS, the insensitive lucigenin-based chemiluminescence detects mainly extracellular superoxide, whereas the fluorescent dye MitoSoxRed detects mitochondrial superoxide. Both DCF-DA and MitoSoxRed detected a small but significant intracellular ROS signal in both normal and X-CGD PMNs (Fig. 5C, D). These data are consistent with the fact that pyocyanin reacts with intracellular NAD(P)H directly. Using lucigenin the detected basal superoxide production in normal PMNs was further increased by pyocyanin (Fig. 5E). In contrast, in X-CGD PMNs basal superoxide release detected with lucigenin was completely absent and pyocyanin failed to increase it (Fig. 5E). To gain insight into the requirement of intracellular and/or extracellular ROS production for pyocyanin-induced NETs, we next measured superoxide production and NET release in the presence of the extracellular ROS scavengers, catalase and superoxide dismutase (SOD). Catalase- and SOD-treatment entirely blocked pyocyanin-stimulated (but not basal) ROS production and NET release (Fig. 5F). These data indicate that although pyocyanin produces superoxide by direct oxidation of NAD(P)H, extracellular superoxide mediating NET release in pyocyanin-exposed neutrophils comes from the activated NADPH oxidase.

### CGD Neutrophils Show Impaired NET Formation in Response to Pyocyanin

**Figure 6:**
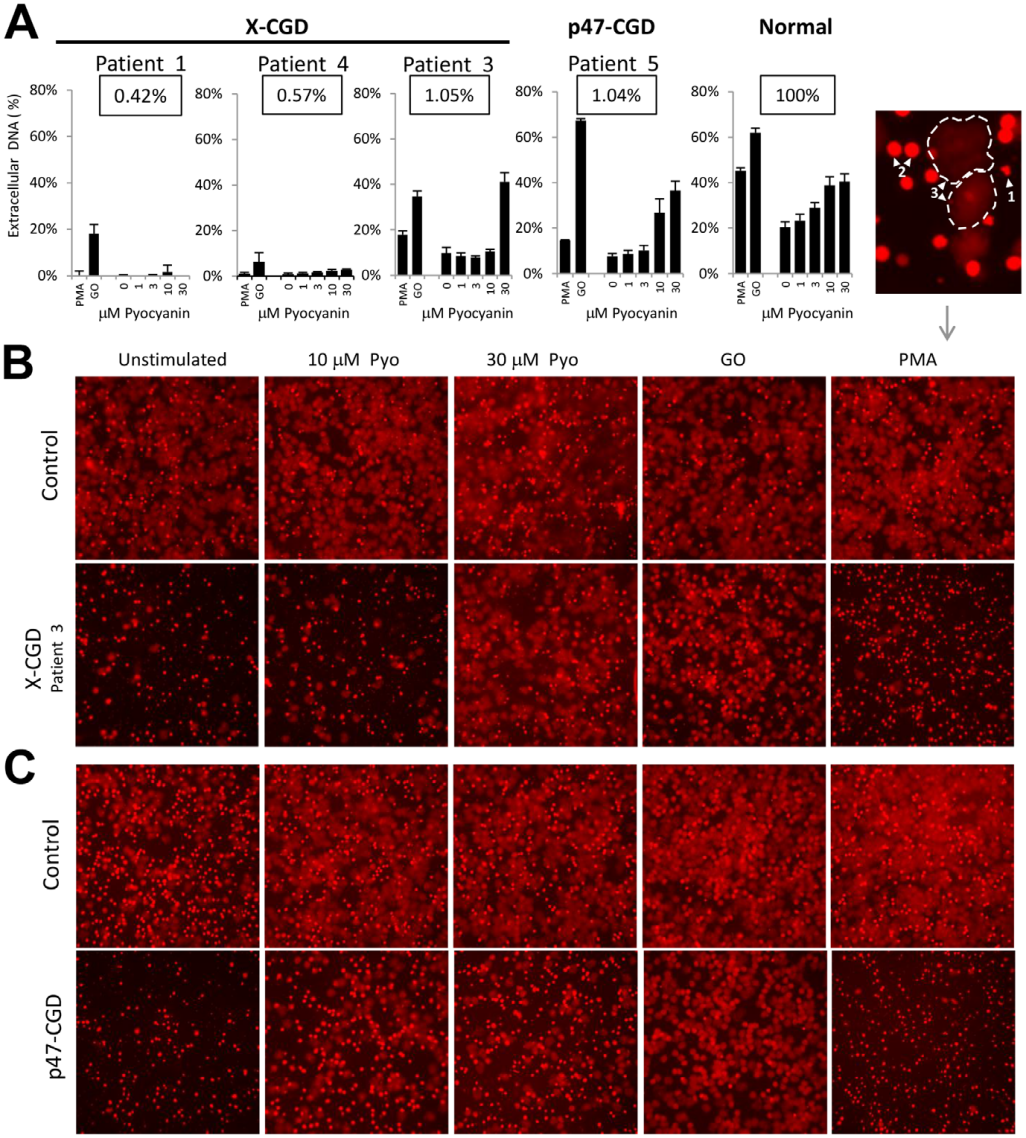
CGD neutrophils have impaired NET formation by pyocyanin. A) Comparison of pyocyanin-induced NET formation among three X-CGD patients, one p47-deficient CGD patient and their normal controls. Adherent non-CGD or CGD neutrophils (patients 1,3,4, 5) were exposed to 0–30 µM pyocyanin, 100 nM PMA or 0.1 U/mL glucose oxidase (GO), Sytox Orange fluorescence was monitored for 3.5 hrs and NET formation was quantified. The numbers in the boxes show residual superoxide productions of CGD neutrophils as % of the average of normal donors (100%). B) Images of adherent neutrophils of CGD patient 3 and a non-CGD control (Sytox Orange fluorescence) after 3.5 hour-exposure to 10, 30 µM pyocyanin, 0.1 U/mL glucose oxidase (GO) or 100 nM PMA. C) Images of NETs formed by p47-deficient CGD neutrophils and normal controls exposed to the same stimuli as previously. Magnified micrograph in the upper right corner shows normal neutrophils in different stages of NET formation at the end (3 hrs) of the Sytox Orange microplate assay. Examples for different stages are marked by white arrowheads and numbers. 1– These cells did not produce NETs. At 3 hrs their plasma membrane became somewhat permeable and Sytox Orange stains their resting multilobular nucleus. 2- These cells are in an intermediate phase. The plasma membrane is permeable, the typical lobulated nuclear morphology is lost but the integrity of the cell is still observed. 3– PMNs in the final stage of NETosis. The nuclear and cell morphology is completely lost, the DNA was released around the cell and it covers a larger area than the original cell size. Due to the weak fluorescent signal of NETs a longer exposure time had to be chosen and PMNs in stages 1 and 2 are overexposed. PMA, phorbol-myristate-acetate; PMN, polymorphonuclear neutrophil.

Next we studied the question of whether the NADPH oxidase mediates pyocyanin-stimulated NET formation as well. To address this we exposed adherent PMNs obtained from four different CGD patients (patient 1,3,4, and 5) and healthy individuals to 0–30 µM pyocyanin, PMA or GO for 3.5 hrs and measured NET release (Fig. 6A–C). A longer time interval was chosen to detect spontaneous NET formation in CGD PMNs. Normal PMNs released 28.4% NETs without stimulation, which was further increased up to 44.2% by 30 µM pyocyanin and 46.3% by PMA (Fig. 6A). Fig. 6B–C show corresponding images of Sytox Orange-stained normal PMNs at the end of the assay. Three subjects studied are X-CGD patients while the fourth one (patient 5) is p47phox-deficient. Although all patients suffer from CGD, their residual superoxide production is different ranging between 0.42–1.05% of that of normal controls. [6] We found that NET formation among the four patients was different. NET release was completely undetectable in patient 1 whose cells have the lowest superoxide production (0.42%) (Fig. 6A). PMNs from patient 4 show minor increase in superoxide production (0.57%) and in basal (0.9%), PMA-stimulated (1.1%) and pyocyanin-elicited (2.8%) NET release, as well (Fig. 6A). Strikingly, patient 3 and 5 who have higher residual superoxide production (1.05%, 1.04%) showed enhanced levels of basal (9.7%, 7.6%) and PMA-stimulated NET formation (17.9%, 14.6%) (Fig. 6A, C). Patients 1 and 4 did not release NETs in response to any pyocyanin concentration tested (Fig. 6A). In the case of patient 3, lower concentrations of pyocyanin did not induce NETs, but the highest dose (30 µM) made PMNs form NETs (41.1%) (Fig. 6A, B). PMNs of patient 5 showed increased sensitivity towards pyocyanin, in which not only 30 µM but also 10 µM pyocyanin induced NETs (Fig. 6A,). These results indicate that basal, pyocyanin- and PMA-stimulated NET release in CGD neutrophils is increasing in relation to residual NADPH oxidase activity.

## Discussion

Neutrophil extracellular trap formation is a fascinating recently discovered mechanism by which neutrophils entrap bacteria. [1] NETs capture *Aspergillus nidulans*, *Candida albicans, Shigella flexneri, Staphylococcus aureus, Salmonella typhimuri*um, nontypeable *Heamophilus influenzae* and Group A Streptococci. Recently, *Pseudomonas aeruginosa*, the major CF pathogen, has also been shown to trigger NETs. [35], [36] However, Pseudomonas-triggered NET formation by adherent neutrophils has not been characterized and the mechanisms involved have been unclear.

Most of the CF clinical isolates of Pseudomonas we surveyed produced pyocyanin (Fig. 1). Most of CF patients’ sputum samples contain pyocyanin levels higher than 10 µM, a concentration that increases spontaneous NET formation by 120% (Fig. 2C) [18], [19], [24]. Pyocyanin is essential for full Pseudomonas virulence in a variety of animal models. [16] Pyocyanin is a phenazine exotoxin [37] with toxic effects in a broad range of target cells that are based on its ability to induce oxidative stress. [23] Although pyocyanin has been studied in several laboratories worldwide, and increased levels of both pyocyanin and neutrophil components in the CF lung have been associated with poor lung function, no reports to date have described any direct effects of pyocyanin on NET formation. Contrary to earlier reports showing that pyocyanin induces apoptosis in neutrophils, we found that pyocyanin in shorter time frames and at lower concentrations (typical for CF airways) induces NET formation (Fig. 2 and 3). [25] Antioxidant and inhibitor treatments revealed roles for ROS, JNK, and PI3K in pyocyanin-elicited NET formation (Fig. 4A–F). We are the first to highlight a role of JNK in NET formation, whereas PI3Ks play an important role in regulating neutrophil functions such as NADPH oxidase assembly and affect NET induction by other agonists such as PMA. [38] The PI3K/Akt/mTOR pathway regulates autophagy in several cell types and autophagy has been implicated in NET formation. [4], [39] ROS produced by NADPH oxidases have been associated with antibacterial autophagy. [40], [41] Since our data show PI3K activation and ROS production by pyocyanin, it is possible that the toxin induces NET formation through autophagy.

Pyocyanin is a redox-active toxin that shuttles electrons between donors and acceptors, thereby catalyzing redox reactions in the host cell. Under aerobic conditions, molecular oxygen is the primary electron acceptor [42] and its reduction by pyocyanin results in production of superoxide anions by one-electron transfer. [22] The main electron donor is NADPH (Fig. 5A). Pyocyanin is a non-enzymatic NAD(P)H oxidase (Fig. 5G) and thus executes the same chemical reaction as the members of the Nox NADPH oxidase enzyme family (Nox 1–5 and Duox 1 and 2). [43] This could be explained by the similarity in chemical structures of pyocyanin and the core of the electron transport chain of every NADPH oxidase, their essential redox cofactor flavin adenine dinucleotide (FAD) (Fig. 5G). In PMNs, the Nox2-based NADPH oxidase is by far the most abundant and most important oxidase involved in crucial cell functions (respiratory burst, intracellular killing, NET formation). Under non-physiological conditions (maximal NADPH oxidase activation by PMA-stimulation), high concentrations of pyocyanin (50–100 µM) inhibit oxidase activity by consuming the substrate, NADPH; in contrast, our current study demonstrates that lower concentrations of pyocyanin (1–30 µM) stimulate NADPH oxidase activity in PMNs (Fig. 2E, 4A, B, E, 5B, E, F). [22] This pyocyanin-mediated superoxide release by PMNs is entirely DPI-sensitive and is absent in CGD PMNs (Fig. 5B, E). [22] We also found that basal, pyocyanin- or PMA-stimulated NET formation in CGD patients was greatly impaired (Fig. 6). Most of the toxic effects of pyocyanin described to date are attributed to the oxidative stress resulting from direct consumption of NADPH. We are the first to show here a new consequence of exposure to pyocyanin (NET formation) that is caused by oxidative stress not derived from direct NADPH oxidation but by activation of the phagocytic NADPH oxidase. Previously we have shown that higher doses of pyocyanin inhibit activation of another NADPH oxidase, Duox in airway epithelial cells. [22] The reason for the opposite effects of pyocyanin on two different NADPH oxidases could be related to the different cell types studied, different toxin doses used, or the relative amounts of pyocyanin and the NADPH oxidases compared to cellular NADPH pools. Further studies are required to unravel the exact mechanism.

Most of the ROS data presented in our study measured superoxide production in neutrophils because superoxide anions are the primary products of both NADPH oxidase activity and direct oxidation of NAD(P)H by pyocyanin. However, the exact molecular identity of ROS directly responsible to initiate NET formation remains to be studied. MPO-derived hypochlorous acid was found to be involved in NET release but no clear evidence for the involvement of hydrogen peroxide, superoxide or mitochondrial ROS has been presented yet. Most likely hydrogen peroxide plays an important role in NET initiation and superoxide is only the primary short-lived ROS product since hydrogen peroxide is relatively long-lived among ROS, can penetrate biological membranes, is readily formed from superoxide by dismutation and when added exogenously (by the glucose/GO system) it triggers maximal NET release [3].

By comparing neutrophils from 4 different CGD patients, we found that basal and PMA-stimulated NET formation was dependent on residual NADPH oxidase activity (Fig. 6). Interestingly, NETs induced by an external ROS source (glucose/GO system) were also dependent on the residual capacity of CGD neutrophils to produce ROS (Fig. 6A). We also showed that higher residual oxidase activity results in lower pyocyanin levels required to induce NETs in CGD PMNs (Fig. 6). These exogenous sources of ROS can trigger NET formation but they are not equivalent to and cannot compensate for some critical level of NADPH oxidase-derived ROS. These observations can be explained by a direct stimulatory effect of ROS on NADPH oxidase activation. NADPH oxidase activation triggered by exogenous hydrogen peroxide was described recently by two groups; one study showed H_2_O_2_ promotes membrane translocation of p40phox, whereas the other showed that H_2_O_2_ affects Ca^2+^ influx and cAbl kinase acting upstream of PKC-delta. [44], [45] Thus, there appears to be some critical “threshold” for NADPH oxidase-derived ROS required to induce NETs and this can be reached more readily in cells already exposed to oxidative stress through basal NADPH oxidase activation. This is the first study to show that NET formation among CGD patients can differ and depends on their residual respiratory burst activity. In the past we have shown the importance of residual superoxide production in neutrophil bacterial killing against *Staphylococcus aureus*. [46] Another recent study linked residual respiratory burst activity and patient survival in a large cohort of CGD patients. [6] According to our findings, the increased survival of CGD patients with higher residual oxidative responses could in part relate to a higher capacity for NET release. [47].

CGD patients, whose neutrophils are unable to make NETs, are not susceptible to infections by Pseudomonas; this may relate to the importance of other oxidases (i.e. airway epithelial Duox) or non-oxidative mechanisms in controlling airway Pseudomonas infections. [22], [23], [43] In cystic fibrosis airways Pseudomonas resides in biofilms protected from neutrophil phagocytic attack or from becoming ensnared in NETs. Thus NET formation may not reflect a critical mechanism for combating *Pseudomonas.* Rather, we speculate that enhanced ROS-dependent NET release by Pseudomonas and pyocyanin contributes to the inflammatory conditions observed in CF airways that are chronically infected.

## Methods

### Human Subjects/ethics Statement

Human subjects included normal volunteers and X-linked (gp91phox-deficient) and autosomal (p47phox-deficient) chronic granulomatous disease patients participating in a study titled “Evaluation of Patients with Immune Function Abnormalities” (National Institute of Allergy and Infectious Diseases/NIH institution review board approved protocol NIH#05-I-0213), and also cystic fibrosis patients participating in an observational study of CF lung disease severity, “Genetics of CF Lung Disease” (Seattle Children's Hospital institutional review board approved protocol 10855 and Partners Healthcare Systems/Massachusetts General Hospital institutional review board approved protocol 2011P000544). The protocols and informed consent procedures were approved by the Institutional Review Boards of the NIAID/NIH, Seattle Children's Hospital, and Partners Healthcare Systems/Massachusetts General Hospital, and the University of Georgia, and the studies were conducted in accordance with the ethical guidelines of Declaration of Helsinki. Human subjects recruited under the guidelines of IRB-approved protocols (NIAID, NIH#05-I-0213 and University of Georgia, UGA# 2012-10769-4) provided written informed consent for participation in the specific studies described below (i.e., neutrophil NADPH oxidase-related functional assays). Human subjects recruited under the guidelines of the Seattle Children's Hospital IRB-approved protocol 10855 provided written informed consent for storage of specimens and data for use in future cystic fibrosis research (i.e., specimen and data banking). Written informed consent for storage of specimens and data for future research use was received from parents or legal guardians of those patients who were minors. The Partners Healthcare Systems/Massachusetts General Hospital IRB assumed regulatory responsibility for a portion of the “Genetics of CF Lung Disease” study after the principal investigator relocated from Seattle Children's Hospital to Massachusetts General Hospital, and reviewed and approved the use of previously stored specimens and data for the specific studies described below, under 45 CFR46.110 and 21 CFR56.110 (expedited review of minimal risk human subjects research). All CF patients in this study were homozygous for the F508del allele of the cystic fibrosis transmembrane conductance regulator (CFTR) gene, and were categorized as having “mild” or “severe” lung disease if they were in the highest or lowest quintile for age of airway obstruction, as assessed by their median forced expiratory volume during the initial second of exhalation. Anticoagulated whole blood (10 mL) was drawn from CGD patients and was processed in parallel with healthy volunteer’s blood. Four X-CGD patients (Patient #1-4) and one p47phox-deficient CGD patient (#5) participated in our study; all subjects were characterized in previous studies with regards to their genetic defects and residual NADPH oxidase activities. [6] Patient #1 (gp91-91a) has the lowest residual superoxide production of 0.94 nmol/10^6^ cells/hr followed by patient #4 (1.29 nmol/10^6^ cells/hr, gp91-22a, 0.57%), patient #2 (1.7 nmol/10^6^ cells/hr, gp-146, 0.75%) and patient #3 (2.38 nmol/10^6^ cells/hr (1.05%). [6] The p47phox−/− patient (Patient #5; p47-16) had NADPH oxidase activity of 2.34 nmol/10^6^ cells/hr (1.04%) and superoxide production in healthy neutrophils was 226 nmol/10^6^ cells/hr (100%). [6].

### Isolation of Human Neutrophils

Neutrophils were purified as described with some modifications. [48] Whole blood was drawn at the Transfusion Medicine Branch of the National Institutes of Health or at the Health Center of University of Georgia. 50 mL blood was anticoagulated by ACD (anticoagulant citrate dextrose) or heparin. 6 mL aliquots of blood were carefully layered on top of 6 mL Histopaque 1119 (Sigma) and centrifuged (800 g 30 min RT). The upper plasma layer was aspirated, and the middle phase containing white blood cells was collected and washed in PBS. Pellets were resuspended in 4 mL PBS and 2 mL were layered on top of a 5-step Percoll gradient (65, 70, 75, 80 and 85%, Sigma) in 15 mL conical tubes. After centrifugation the 70–75–80% Percoll layers containing neutrophils were collected and washed in PBS. Pelleted neutrophils were resuspended in autologous serum, cell concentrations were determined and cells were kept at room temperature until use. Viability of the cells was determined by Trypan Blue dye extrusion and resulted in >98% viable neutrophils. The purity of the preparations was determined by Wright-Giemsa staining and yielded >95% neutrophil granulocytes.

### Pseudomonas Aeruginosa Strains

PA14 wild-type and pyocyanin-deficient mutants PhzM were provided by Frederick M. Ausubel (Harvard Medical School, Boston). Clinical isolates of *Pseudomonas aeruginosa* were cultured from sputum or oropharyngeal swabs obtained from CF patients participating in the “Genetics of CF Lung Disease” observational study. Isolates were categorized as “early” or “late” relative to the course of each patient’s onset of infection. Early isolates were obtained at 3 months to 11 years of age; late isolates were obtained at 5–20 years later than early isolates. All clinical isolates were stored in Luria-Bertani broth with 16% glycerol at −80°C.

### Determination of Pyocyanin Concentration

CF clinical isolates of *Pseudomonas aeruginosa,* the pyocyanin-producing control strain PA14 WT (wild-type) and the pyocyanin-deficient strain PA14 PhzM were grown in LB liquid medium for 48 hrs with continuous shaking. Bacteria were removed by high-speed centrifugation and pyocyanin concentrations in the culture supernatants were determined by subsequent chloroform/0.2 M HCl extraction steps. Absorbance was measured at 520 nm and concentration values (micromol/L) were calculated using a calibration series with known pyocyanin concentrations. The experiment was repeated three times and the mean+/−S.E.M. values are presented (Fig. 1). The clinical isolates were deidentified during the experiments and were only unblinded once the results were obtained. Pyocyanin was purchased from Cayman Chemical and dissolved in DMSO as 20 mM stock. Final concentrations were diluted in HBSS and were free of bacterial LPS as measured by the LAL endotoxin test kit.

### Visualization of NETs by Fluorescence Microscopy

To visualize NETs released by pyocyanin-stimulated neutrophils, 5×10^5^ neutrophils were allowed to adhere in 2 mL assay medium to poly-d-lysine-coated 35 mm glass bottom culture dishes (MatTEK Corp) (30 min, 37°C). Pyocyanin (1, 3, 10 or 30 µM) was added to stimulate NET formation. After three hours, DAPI, Sytox Orange (2.5 µM) and/or Sytox Green (5 µM) were gently added and the samples were immediately analyzed without further disturbance with a Leica DM IRBE inverted fluorescence microscope. Images were taken, analyzed and processed with SPOT Advanced software (Spot Imaging Solutions, Sterling Heights, MI). To visualize pyocyanin-triggered NET formation, fluorescence images were also recorded on Sytox Orange-stained neutrophils studied in the 96-well plate assay with the Leica fluorescence microscope.

### Quantification of Neutrophil Extracellular Traps

25,000 neutrophils/well were allowed to adhere on uncoated 96-well black transparent bottom plates at 37°C in 50 µL/well assay medium (FBS- and phenol red-free RPMI 1640 medium containing 1% HEPES and 0.1% human serum albumin (HSA)). After 15 minutes, 50 µL/well HSA-free assay medium containing 5 µM Sytox Orange (Invitrogen) membrane-impermeable DNA dye (final cc: 2.5 µM) and stimuli or inhibitors were added gently. Fluorescence (excitation: 530 nm, emission: 590 nm) was recorded in fluorescence plate reader (Labsystems, Fluoroskan) for 3 hours at 2 min intervals (37°C, no shaking). The initial fluorescence in samples containing neutrophils and 0.5 mg/mL saponin was taken as maximal signal (100%). Relative fluorescence change in the unknown samples over the 2.5–3.5 hr period was calculated and referred to as NET formation (% of max). We used uncoated plates to promote spontaneous NET formation. When bacteria were used to induce NETs, PA14 wild-type and pyocyanin-deficient PhzM strains were grown overnight in LB medium. Bacteria were washed twice in HBSS and were added to adherent neutrophils (2×10^6^ PA14/well) in HBSS. When inhibitors were applied the following concentrations were used: SB202474 (negative control for SB compounds, Sigma, 10 µM), SB600125 (JNK inhibitor, Sigma, 10 µM), wortmannin (PI3K inhibitor, Sigma, 100 nM), PD98059 (MEK1 inhibitor, Sigma, 25 µM), SB203580 (p38 inhibitor, Sigma, 10 µM). The ROS scavenger N-acetyl-cysteine (NAC) was dissolved at 1 M concentration in HBSS, the acidic pH was adjusted to 7.4 and NAC was used in the experiments at a final concentration of 10 mM.

### Immunostaining of Myeloperoxidase (MPO) and Citrullinated Histone H4 (citH4)

2×10^5^ neutrophils/well in 1 mL assay medium were allowed to adhere to sterile 13 mm round glass cover slips placed in 12-well plates for 30 min. Pyocyanin was added to the cells in 100 uL assay medium resulting in 20 µM final concentration. Neutrophils were incubated for 3 hours at 37°C, then fixed by 4% paraformaldehyde dissolved in PBS for 10 min. Cells were permeabilized in 0.1% Triton X-100 for 2 min RT, washed 3 times in PBS for 5 min and blocked with 5% donkey serum in PBS for 30 min 37C. Neutrophils were incubated with monoclonal mouse anti-human myeloperoxidase/FITC antibody (Dako, Clone MPO-7) (2 hrs, RT, dark, 1∶1000), or polyclonal rabbit anti-histone H4 (citrulline 3) (Millipore, 1∶1000), washed 3 times in PBS. After Alexa Fluor 488-labelled goat anti-rabbit secondary antibody was added (1 hr, 1∶1000), cells were stained with DAPI (2 min, RT, 1∶10000) and washed 3 times in PBS. Preparations were mounted with ProLong Antifade Kit (Molecular Probes) following the manufacturer’s instructions and analyzed with a Leica fluorescence microscope. Fluorescence images were analyzed with SPOT Advanced software. The original green fluorescence of the histone-staining was converted in the imaging software to red to illustrate the co-localization of DNA and citH4 (Fig. 3B).

### Measurement of Superoxide Production

Superoxide production in neutrophils was measured either by Lucigenin (9,9′-Bis-N-methylacridinium nitrate)-amplified chemiluminescence or by the Diogenes cellular luminescence enhancement system (National Diagnostics, Atlanta, GA, USA). Lucigenin detects both, intracellular and extracellular superoxide but it is insensitive, whereas Diogenes only measures extracellular superoxide but it is highly sensitive. In both assays the cells were incubated in 96-well white plates and luminescence was followed in a Luminoskan Ascent microplate luminometer (ThermoScientific, Hudson, NH, USA). Superoxide production is either presented as kinetics of luminescence or as integrated luminescence units (int. RLU, area under the measured curve over specified time intervals).

Neutrophils were incubated with 50 µg/mL Lucigenin in HBSS for 10 min at 37°C before addition of any stimuli. To measure pyocyanin- or PMA-stimulated superoxide production, neutrophils (10^6^/mL) were treated with 1 µM PMA or 1–100 µM pyocyanin and luminescence was recorded for 30 min at 37°C.

Superoxide production was also measured by the Diogenes cellular luminescence enhancement system (National Diagnostics, Atlanta, GA, USA). Neutrophils in suspension (10^6^/mL, 10 min) or attached (50,000/well, 1 hr) were stimulated with 1 µM PMA or 1–30 µM pyocyanin and luminescence was recorded at 37°C.

Superoxide production by 50 µM pyocyanin in a cell-free system in the presence of 20 µM β-NADH or β-NADPH was measured for 30 min with the Diogenes assay.

### Measurement of Intracellular ROS Production

Human neutrophils (normal and X-CGD) were incubated with 1 µM DCFH-DA (2′-7′-Dichlorodihydrofluorescein diacetate) for 10 min, washed twice in HBSS and exposed to 1 µM PMA or 20 µM pyocyanin. After 30 min ROS-mediated oxidation of H_2_DCF into the highly fluorescent DCF was measured by flow cytometry (AlexaFluor 488, 20.000 cells/sample). Data are shown as histograms.

### Measurement of Mitochondrial Superoxide

Neutrophils (normal and X-CGD) were incubated with 5 µM MitoSox Red (15 min 37C), washed twice and stimulated either by 30 µM pyocyanin or 1 µM antimycin A. After 30 min mitochondrial superoxide production was detected by flow cytometry (exc/em: 510/580 nm).

### Statistical Analysis of Data

Data are shown as mean +/− SEM. When independent variables were studied significance was calculated with Student’s t-test. When results of trends were compared data were analyzed by ANOVA and *post-hoc* Dunnett’s test. Significant changes were marked as * when p<0.05, ** when p<0.01 and *** when p<0.001.
